# Editorial: Advancing therapeutic strategies for relapsed/refractory acute lymphoblastic leukemia

**DOI:** 10.3389/fmed.2026.1791558

**Published:** 2026-02-09

**Authors:** Daniel Ebrahimi-Fakhari, Athanasios Tragiannidis

**Affiliations:** 1 Pediatric Hematology and Oncology, University Hospital Münster, Münster, Germany; 2 Pediatric and Adolescent Hematology Oncology Unit, 2nd Pediatric Department, Faculty of Health Sciences, Aristoteleio Panepistemio Thessalonikes, Thessaloniki, Greece

**Keywords:** ALL, Bcl-2, pediatric, Philadelphia chromosome (BCR-ABL), prognosis, proteomics, relapsed/refractory (R/R), targeted therapy

Relapsed and refractory hematologic malignancies remain among the hardest clinical problems in contemporary oncology: durable remissions are increasingly achievable, yet relapse, treatment resistance and failure, and therapy-related toxicity still limit long-term survival. The four papers collected under this Research Topic advance complementary pieces of a single puzzle—how to translate disease biology into safer, more effective, and more personalized care for patients who relapse or fail first-line therapy. Together they illustrate three pragmatic priorities for the field: (1) smarter, biology-driven bridging and salvage regimens; (2) robust, minimally invasive disease monitoring; and (3) evidence from real-world and regional cohorts to guide implementation.

A first thread links targeted, low-toxicity combinations to effective bridging before definitive therapy. Jin et al. report a compelling case in which a venetoclax–chidamide–azacitidine (VCA) regimen induced rapid complete remission of extramedullary MLL-AF4 B-ALL and served as an effective bridge to CD19 CAR-T therapy, with complete metabolic remission thereafter. The case highlights how rational combinations that target anti-apoptotic signaling and epigenetic dysregulation can control aggressive, transplant-refractory disease while preserving performance status for subsequent cellular therapy.

A second, related contribution evaluates Olverembatinib in relapsed or MRD-persistent Philadelphia-chromosome-positive ALL Jiang et al.. The study reports manageable toxicity in this real-world cohort and suggests Olverembatinib can be incorporated into future strategies for TKI-resistant Ph+ disease.

Taken together, the two aforementioned publications underline a key point: newer targeted agents and biologically rational combos could convert otherwise hopeless relapses into states amenable to consolidative curative therapy.

Third, sensitive and practical monitoring tools are needed to detect relapse earlier and tailor interventions. Wu et al. present proteomic discovery and ELISA validation that identify thrombospondin-1 (THBS1) and lactoferrin (LTF) as serum proteins significantly downregulated in relapsed/refractory multiple myeloma.

Larger validation and prospective testing will be required, but the study illustrates how high-throughput proteomics can yield clinically actionable signatures for relapse monitoring.

Finally, robust epidemiology and cohort studies remain essential to translate advances into population-level benefit. Zhang et al.'s comparative analysis of pediatric lymphoblastic lymphoma highlights substantial differences in stage at presentation, use of radiotherapy, and period-specific survival—some of which may reflect pandemic-related care disruption. Their work reminds clinicians and trialists that biologic advances must be considered within the realities of staging patterns, treatment access, and health-system stresses that vary by region and era; these factors influence both outcome and how new therapies should be implemented.

Taken together, these four contributions chart a coherent agenda for the next phase of progress in relapsed/refractory hematologic malignancies:

- Prioritize biology-first bridging strategies that reduce tumor burden with tolerable toxicity (targeted agents, epigenetic modifiers) so patients can receive curative consolidation (cellular therapy or transplant).
- Invest in minimally invasive monitoring (proteomic signatures, circulating markers, refined MRD assays) to detect relapse earlier and trigger preemptive salvage.
- Build multinational, real-world datasets to understand regional differences in presentation, access, and outcomes and to ensure equitable implementation of new regimens.
- Design prospective trials that combine these elements: biomarker-guided entry, biology-matched bridging, and endpoints that include functional outcomes (ability to receive CAR-T/allo-HCT), not just short-term response.

Limitations are intrinsic to the contributions: single-patient case reports and retrospective series cannot establish efficacy definitively, and proteomic biomarkers need large, prospective validation. Still, by linking mechanistic rationale (why a regimen should work), pragmatic endpoints (bridging to curative therapy), and population context (who will benefit and under what health-system constraints), the collection models how translational hematology can move from isolated successes to scalable improvement.

We hope these articles motivate coordinated pipelines: rapid biological characterization at relapse, adaptive low-toxicity regimens to lower burden, sensitive blood-based monitoring to time interventions, and multicenter trials that measure both molecular and patient-centered outcomes. The future of relapsed/refractory leukemia and other hematologic malignancies lies not in any single silver bullet, but in integrated, biology-informed care pathways—precisely the direction these papers point toward.

